# Interactions between long-acting antiretrovirals and opioids: a call for clinical awareness

**DOI:** 10.1128/cmr.00395-25

**Published:** 2026-03-12

**Authors:** Candy Carbajal, Florida Owens, Dileepkumar Veeragoni, Myosotys Rodriguez, Francisco Fernandez-Lima, Adel Nefzi, Shilpa Buch, Nazira El-Hage

**Affiliations:** 1 Department of Cellular and Molecular Medicine, Herbert Wertheim College of Medicine, Florida International University 5450, Miami, Florida, USA; 2 Department of Chemistry and Biochemistry, College of Arts and Sciences, Florida International University 5450, Miami, Florida, USA; 3 Center For Translational Science, Florida International University 5450, Miami, Florida, USA; 4 Department of Pharmacology and Experimental Neuroscience, University of Nebraska Medical Center 12284, Omaha, Nebraska, USA; University of California San Diego, La Jolla, California, USA

**Keywords:** long-acting antiretrovirals, opioids, HIV, drug-drug interactions, metabolic enzymes, drug transporter

## Abstract

This review summarizes potential drug-drug interactions (DDIs) between long-acting antiretrovirals (LAAs) and opioids, focusing on pharmacodynamics (PD), pharmacokinetics (PK), and side effects related to absorption, distribution, metabolism, and excretion (ADME). It also covers dysregulation in epigenetics and in the immune system. A comprehensive literature review was performed using PubMed and Google Scholar, along with data from the Liverpool HIV Drug Interactions Database, to evaluate pharmacokinetic interactions between LAA drugs, such as cabotegravir, rilpivirine, lenacapavir, and dapivirine, with opioids used in clinical settings, including morphine, tramadol, oxycodone, and codeine; medications for opioid use disorder (MOUD), such as methadone and buprenorphine; and the illicit opioids, including fentanyl and heroin. The review highlights key factors increasing DDI risk and discusses clinical considerations for managing preexposure prophylaxis (PrEP) or antiretroviral therapy (ART) in persons who use opioids. Lastly, it proposes research strategies for studying DDIs, including the use of animal models, physiologically based pharmacokinetic (PBPK) models, and clinical trials.

## INTRODUCTION

Human immunodeficiency virus (HIV) infection remains a significant challenge in modern medicine (1, 2). A cure or effective vaccine for HIV remains out of reach due to the virus’s high variability and its ability to integrate its genome into host cells, leading to lifelong infection and serious health risks. The presence of HIV alongside other conditions, referred to as comorbidities, further complicates treatment, leading to poorer health outcomes. In recent years, substance use has significantly increased in the United States, particularly due to the opioid crisis, which has been declared a public health emergency. Of the 11 million people who inject drugs globally, an estimated 1.4 million are living with HIV (3). Approximately 10% of new HIV infections are associated with injection drug use, especially opioids (4). These statistics demonstrate an urgent need for effective prevention strategies. Long-acting injectable antiretroviral therapy (LAA) is the recently approved approach for HIV prevention and treatment, showcasing a novel mechanism of action and prolonged persistence of effective drug concentrations (5, 6). An intramuscular (IM), subcutaneous (SC), or intradermal (ID) injection of a suspension or lipophilic solution creates a drug “depot” from which the drug is released over an extended period (7–9). People living with HIV (PLWH), including those with comorbid opioid use disorder (OUD), are prioritized for the use of LAA therapies, either as preexposure prophylaxis (PrEP) or antiretroviral therapy (ART) (10, 11). Currently, the FDA has approved three LAA options: cabotegravir (CAB), rilpivirine (RVP), and lenacapavir (LEN) (12, 13). Additionally, other long-acting but not injectable ART are in phase III development for HIV prevention and treatment, such as dapivirine (DPV), as a vaginal ring (14). LAA offers a significant shift in therapy from daily pills to infrequent injections, overcoming the physical, emotional, and psychosocial difficulties of the oral regimen. This innovative approach is a promising tool to effectively prevent and control HIV infection and help reach the global goal of ending the HIV/AIDS epidemic as a public health threat by 2030.

Polysubstance use is widespread in PLWH and has long been linked to drug-drug interactions (DDIs) (15–17). Recent evidence confirms a significant rise in stimulant use, such as cocaine and methamphetamine across the U.S.A., which is heavily compounded by polysubstance use involving potent synthetic opioids like fentanyl (18–20). These practices can affect the ART through biological interactions, behavioral barriers to adherence, and increased risk of drug-related toxicity. Additionally, opioids used in clinical settings, such as morphine and codeine, and medications for opioid use disorder (MOUD), such as methadone and buprenorphine, may also be affected by DDIs. Polysubstance use can lead to unpredictable effects, and some combinations have more potent effects than either drug alone. For example, mixing codeine with other medications could significantly increase sedation and the risk of severe adverse effects. To address this issue, it urgently needs more access to harm- or risk-reduction strategies designed to prevent overdoses among these populations, including checking programs, education, and stigma reduction (21).

ART features a wide range of pharmacological outcomes. However, individual drugs differ significantly in safety, tolerability, PK profiles, and interactions with other drugs, such as opioids and MOUD (22). The risk of drug failure, toxicity, or overdose, which cannot be easily reversed due to the narrow therapeutic index of opioids and MOUD and the nature of the LAA, must be avoided. Factors such as comorbidities, body mass index (BMI), sex, genetics, and polydrug use can interfere with the drug metabolism of these medications (5, 6). Therefore, as a new therapy, it presents unique challenges that need to be addressed, including potential uncertainties from the prolonged presence of measurable drug levels after a single injection and the unusual route of administration. Variations in drug response due to DDIs can impact both the effectiveness and side effects of these medications, increasing the risk of drug resistance and toxicity (22–26).

The phase I metabolic enzyme, cytochrome P450 (CYP), particularly CYP3A4, along with the phase II enzyme system, uridine diphosphate glucuronosyltransferase (UGT), is responsible for metabolizing many drugs, including opioids and antiretrovirals (27–29). Additionally, drug transporters, such as permeability glycoprotein (P-gp), and others that are involved in phase III metabolism play essential roles in the biodistribution of LAA, opioids, and MOUD (25, 30). Since these medications utilize similar enzymatic systems for metabolism and transporters for distribution, they may influence how drugs are absorbed, distributed, metabolized, and eliminated from the body. Effective monitoring, increased patient choice, and shared decision-making are essential for the use of LAI PrEP and ART in individuals with OUD, especially those receiving treatment with buprenorphine and methadone. It is therefore crucial to fully understand the risks associated with these new therapies, especially the potential DDIs in persons who use opioids receiving LAA for HIV prevention and treatment. Some studies have explored concerns about DDIs for LAA and other commonly used drugs in the context of HIV infection, such as antimycobacterials^31^, ^32^; however, the potential interactions between LAA and opioids remain understudied. In this review, we examine the current literature on the clinical pharmacology of LAA, focusing on pharmacokinetics (PK) and pharmacodynamics (PD). It identifies clinically relevant DDIs associated with LAA and opioids, providing critical clinical considerations and guidance for preventing and managing these risks in PLWH and PrEP users. Finally, we summarize the key prospective approaches for evaluating the efficacy, safety, and interactions associated with the concurrent use of LAA and opioids.

## LONG-ACTING ANTIRETROVIRALS

### Cabotegravir (CAB)

CAB is an extended-release integrase strand transfer inhibitor (INSTI) used to treat and prevent HIV infection. Its mechanism of action involves inhibiting the enzyme integrase, which is essential for HIV replication (33). Currently, CAB is administered every 8 weeks through IM injection. It is the first injectable PrEP approved by the U.S. Food and Drug Administration (FDA), and when combined with RPV, it forms a complete regimen prescribed to treat HIV infection in individuals aged 12 and older, replacing their current oral HIV medication (7). The 2021 FDA guidelines recommend prescribing CAB/RPV for PLWH with viral suppression on their oral ART. This group represents 65.1% of PLWH in the United States as of 2023 (2). The intramuscular administration of CAB has increased the feasibility of CAB extending beyond HIV PrEP, as it has been shown to offer meaningful benefits to people who use drugs and those experiencing homelessness, especially for patients having difficulty adhering to oral antiretrovirals (34). Additionally, clinical data on obese individuals with HIV show that using monthly long-acting CAB combined with rilpivirine decreases drug exposure in this group, causing drug levels to fall below the target thresholds at steady state, especially with bimonthly dosing. Therapeutic drug monitoring is advised to help adjust the dosing interval (35). CAB is mainly metabolized in the liver by UGT-1A1, with minor contributions from 1A9, and is primarily excreted in urine, with small amounts in bile and feces (6). A summary of the key PK parameters is provided in Table 1. Because of the very long half-life of CAB, some individuals have detectable levels a year after a single injection. However, these parameters can vary among individuals. A recent study characterizing CAB PK in individuals with and without HIV reported that the absorption rate was 50.9% lower in females and decreased with increasing BMI. Moreover, clearance was 17.4% higher in the setting of tobacco use (36). The HPTN-083 and 084 studies found that CAB LA was very safe and is being used clinically in individuals receiving opioid treatments, including buprenorphine and methadone, with no reports of severe adverse events. However, it is essential to emphasize that monitoring of adverse effects in individuals using CAB-LA should continue through different phases, especially over an extended period. This includes evaluating PK, PD, and signs of toxicity—particularly in people taking medications with a narrow therapeutic index, such as methadone, where small dose increases can lead to respiratory depression, cardiac arrhythmias, or overdose (8).

**TABLE 1:** Characteristics of long-acting antiretroviral and possible interactions with opioids^*a*^

Characteristics	Cabotegravir (CAB)	Rilpivirine (RPV)	Lenacapavir (LEN)	Dapivirine (DPV)
Action’s mechanism	Integrase inhibitor	Non-nucleoside reverse transcriptase inhibitor	Capsid inhibitor	Non-nucleoside reverse transcriptase inhibitor
Function	PrEP and ART	ART	PrEP and ART	PrEP
Administration	Intramuscular	Intramuscular	Subcutaneous	Intravaginal ring (IVR)
Tmax	7 days	3–4 days	77–84 days	1–7 days
Vd	12.3 L	132 L	9,500–11,700 L	Limited data
Half-life	6–12 weeks	13–28 weeks	8–12 weeks	82 h/plasma 13 h/vagina
Metabolizing enzymes	UGT1A1 UGT1A9	CYP3A4	CYP3A4 UGT1A1	CYP3A4 CYP1A1
Transporters	Substrate of P-gp and BCRP Inhibitor of OAT1 and OAT3	Inhibitor of ABC and SLC transporters Inhibitor of OCT	Substrate of P-gp and Inhibitor of P-gp and BCRP	DPV does not act as a substrate or inhibitor
Possible interaction with opioids and other medications	Morphine, codeine, oxymorphone, oxycodone	Fentanyl, heroin, buprenorphine, methadone, oxycodone	Fentanyl, heroin, morphine, methadone, oxycodone	Fentanyl, heroin, morphine, methadone, oxycodone

^*a*^ The table summarizes the pharmacological characteristics of currently available long-acting antiretroviral therapies, including mechanisms of action, route of administration, half-life, key pharmacokinetics and pharmacodynamic properties, metabolic pathways, drug transporters, and known predicted drug-drug interactions with opioids. Potential interactions are based on shared involvement of UGT (UDP-glucuronosyltransferases), cytochrome P450 enzymes, drug transporters, or overlapping effects on central nervous system. PrEP, preexposure prophylaxis; ART, antiretroviral therapy; Tmax, time to maximum concentration; Vd, volume of distribution; L, liter; UGT, UDP-glucuronosyltransferase; CYP, cytochrome P450; BCRP, breast cancer resistance protein; OAT, organic anion transporter; ABC, ATP-binding cassette; SLC, solute carrier transporters; OCT, organic cation transporter; P-gp, permeability glycoprotein.

### Rilpivirine (RPV)

RPV is a non-nucleoside reverse transcriptase inhibitor (NNRTI), whose mechanism of action is non-competitive binding to and inhibition of HIV reverse transcriptase (37). Combined CAB/RPV constitutes the first LAI-ART regimen approved for adults with HIV. This regimen demonstrates high efficacy and safety for maintaining HIV suppression, with studies showing that it is not inferior to daily oral therapy and even superior in some cases, especially for individuals facing adherence challenges (38, 39).

As shown in Table 1, like CAB, RPV exhibits different PK profiles. RPV is highly protein-bound, with about 99.7% binding primarily to albumin. Its elimination is mainly mediated by hepatic metabolism via CYP3A4. A multicenter prospective observational study evaluating RPV concentration and effectiveness reported significant variability in PK. In this study, it was found that over 50% of RPV C trough levels were below the 50 ng/mL target during the 32-week observation period (40). Reports have indicated that RPV could cause side effects such as mild, self-resolving liver issues and rashes (38).

### Lenacapavir (LEN)

The HIV capsid inhibitor LEN is the most recently approved LAA. LEN is a first-in-class inhibitor that targets the HIV capsid, a critical component of the virus life cycle. It prevents the virus from integrating into the host cell DNA (41). Additionally, LEN impairs the production of new virions, significantly inhibiting viral replication at both the early and late stages of the life cycle. LEN is currently approved in multiple countries for treating adults with multidrug-resistant HIV, and it has demonstrated remarkable efficacy, making it a key component of salvage therapy when is combined with other antiretroviral drugs (13). Studies have shown that LEN is well tolerated and achieves high rates of long-term virological suppression.

Furthermore, LEN was recently approved by the FDA as an alternative PrEP option, and its pharmacological features are mentioned in Table 1. Research indicates that both a twice-yearly SC injection and a single once-yearly IM injection of LEN are safe and highly effective for PrEP in diverse populations, reducing the risk of acquiring HIV by 96% (9, 42, 43). The six-month injectable formulation of LEN has been approved for PrEP, and it is currently under evaluation in vulnerable populations, including persons who inject drugs and individuals with opioid use disorder, as part of the PURPOSE 4 trial (HPTN 103). This clinical study is designed to assess the PK of LEN and to evaluate the safety of both LEN and emtricitabine/tenofovir disoproxil fumarate (F/TDF) for PrEP among people who inject drugs (PWID) in the United States. LEN is gradually released from the injection site. It requires an initial phase using oral tablets in combination with the first subcutaneous injection to ensure adequate PK exposure during the early days of treatment. After this phase, SC administration is planned every six months, with a half-life of 8–12 weeks, to maintain drug levels within the therapeutic range (9).

LEN is metabolized by the CYP3A4 isoenzyme of the cytochrome system and undergoes glucuronidation by UGT1A1. It is primarily eliminated via the biliary route and excreted in feces. One study found that, after a single injection of the radiolabeled drug, 76% was excreted in feces and less than 1% in urine. Additionally, unchanged LEN accounted for 69% in plasma and 33% in feces (44). LEN does not induce CYP3A enzymes but could inhibit CYP3A4, potentially increasing the risk of DDIs with other drugs that are substrates of this enzyme system (41). Due to the prolonged presence of LEN, its inhibitory effects could continue to affect the PK exposure of these substrates for up to nine months following the last injection.

### Dapivirine (DPV)

DPV, a non-nucleoside reverse transcriptase inhibitor (NNRTI), is a substituted diarylpyrimidine derivative that irreversibly binds to HIV reverse transcriptase (RT), thereby preventing HIV replication. DPV binds to the RT enzyme, disrupting its structure and function. This action blocks the enzyme’s ability to convert viral RNA into proviral DNA, a crucial step in the HIV replication cycle (45). The dapivirine vaginal ring (DVR) is the first long-acting, non-systemic HIV prevention product approved in several African countries and is under investigation for approval worldwide (14, 46). DVR is a flexible silicone ring inserted into the vagina that gradually releases DPV over a month. Because of its method of use, DVR is currently approved only for individuals with a vagina. It has demonstrated promise in reducing HIV incidence and could be a viable option for cisgender women, especially those at higher risk, such as African, Caribbean, and Black (ACB) women. DVR might play a role in addressing the feminization of the HIV epidemic, as women make up nearly half of all people living with HIV worldwide. However, its limitations include decreased effectiveness in women under 21, potential drug interactions and side effects, and adherence challenges. This underscores the need to combine DVR with comprehensive support and counseling, along with other prevention strategies, such as daily oral or injectable PrEP (14).

DVR exhibits a distinct PK profile (Table 1) characterized by sustained release in the vaginal area, resulting in higher concentrations near the ring and lower systemic concentrations in plasma. However, a slight increase in mean plasma DPV concentrations over time has been observed. Chen et al. reported that the median plasma DPV concentration of 268.0 pg/mL was also similar to the median DPV concentration in reproductive-age women (264 pg/mL) (47). In another study, Noguchi et al. demonstrated that DPV was present in breast milk, but at low concentrations, with a median breast milk-to-plasma ratio of 2-fold based on Cmax (48). DPV is metabolized by CYP450 and UGT enzymes, with CYP1A1 and CYP3A4 identified as the main enzymes. Little to no metabolism occurs via UGT1A and UGT2B7. DPV is eliminated through both renal and biliary pathways, although interactions with certain CYP and UGT enzymes, including those expressed locally in the female reproductive tract, can occur (49). Additionally, DPV was identified as a strong inhibitor of CYP1A1; a moderate inhibitor of CYP1B1, CYP2C8, CYP3A4, UGT1A1, and UGT1A4; and a weak inhibitor of CYP1A2, CYP2B6, CYP2C19, UGT1A3, UGT1A6, UGT1A7, UGT1A9, and UGT2B7. DPV has not demonstrated induction of CYP1A2, CYP2B6, or CYP3A4 enzymes.

## OPIOIDS IN THE CONTEXT OF HIV INFECTION

Opioids are central nervous system (CNS) depressants that primarily decrease pain perception by binding to opioid receptors (mu, delta, and kappa) in the brain, spinal cord, and periphery. This binding triggers a cascade of events that can affect the ascending (afferent) and descending (efferent) pain pathways, influencing how pain signals are transmitted and processed in the nervous system (28). Research indicates that PLWH are at increased risk for chronic pain, are more likely to be prescribed opioid analgesics—often requiring higher dosages of these medications—and have a higher prevalence of substance use disorders (16, 50). Additionally, as these drugs are often administered through injection, individuals who share needles, syringes, or other injection equipment are at a higher risk of contracting or transmitting HIV (51). Furthermore, opioid use is linked to behaviors that further elevate this risk, such as engaging in transactional sex for drugs or money, having multiple sexual partners, and inconsistently adhering to HIV prevention methods or treatment regimens (52). A recent report from UNAIDS establishes that in 2022, the risk of HIV transmission was 35 times higher among people who injected drugs (53). Opioid metabolism occurs in two phases. Phase I involves enzymes that modify the drug through oxidation or hydrolysis, while phase II uses UGT enzymes for glucuronidation, which conjugates the drug with glucuronic acid to enhance its excretion. According to the DrugBank database, eight enzymes are involved in opioid metabolism. These include cytochrome P450 isotypes, such as 3A4, 2B6, 2C8, 2C9, 2D6, and several UGTs, including UGT2B7 and UGT1A1, among others.

Within the context of HIV infection, opioid use can be categorized into three groups: prescribed opioids in medical settings, MOUD, and illicit opioids, whose main characteristics are summarized in Table 2. There is a growing movement toward non-opioid pain treatments, driven by the risks associated with opioids. Medications like gabapentin (and its cousin pregabalin/Lyrica) are key alternatives for nerve pain, along with a push for multidisciplinary approaches. However, the significant increase in the illicit use of these drugs requires their evaluation and careful consideration of the clinical implications.

**TABLE 2:** Commonly used opioids in the context of HIV infection^*a*^

Type of opioids	Use	Administration	Metabolizing enzymes	Transporters	Possible risks in DDIs with antiretroviral (ARV)
Opioids in medical settings					
Morphine	Managing moderate to severe acute and chronic pain in PLWH	Extended-release oral tablets, injectable: IV, IM, SC, and epidural, suppositories, and transmucosal (TM)	Mainly UGT2B7, and others involved UGT1A1, UGT1A3, UGT1A6, UGT1A8, and UGT2B1	P-gp OCT1	Increased toxicity, increased side effects: profound sedation, respiratory depression, reduced ARV effectiveness, and decreased pain relief
Tramadol	Used as a second- or third-line agent for chronic neuropathic pain in PLWH	Oral tablets, IV, IM, SC injection	CYP2D6 and CYP3A4	P-gp OCT1	Potentially respiratory depression, serotonin syndrome, and reduced tramadol effectiveness
Oxycodone	Sometimes used as second- or third-line treatment to control neuropathic pain in PLWH who do not respond to first-line therapies	IV, IM, IN, SC, rectal, epidural, and oral using solutions and tablets	CYP3A4, CYP3A5, and CYP2D6	P-gp	Significantly increased levels, severe side effects like respiratory depression, sedation, overdose, and potentially addiction, toxicity, and reduced pain relief
Codeine	Prescribed for the management of mild to moderately severe pain	Oral tablets, capsule, solution, syrup form, IM, SC injection	CYP2D6 CYP3A4 UGT2B7, UGT1A1, and UGT1A8	OCT1	Increased toxicity, causing overdose symptoms, or reduced pain relief
Medications for opioid use disorder (MOUD)					
Methadone	Used in the management of OUD	Oral tablets	CYP3A4 and CYP2B6	P-gp	Methadone withdrawal due to decreased methadone levels, and risk of accumulation that may lead to delayed toxicity and respiratory depression
Buprenorphine	Used in the management of OUD	Sublingual tablets/films, transdermal patches, and long-acting injections (LAI)	CYP3A4	P-gp BCRP	Generally, has fewer significant drug interactions with ARVs than methadone. However, some specific interactions exist, which may require close clinical monitoring or dose adjustments
Illicit opioids					
Fentanyl	Unregulated use of fentanyl has led to recreational substance use and addiction	IV, IM, IN, transdermal (TD), and TM	CYP3A4	P-gp OAT	Significantly increased blood levels, which can lead to overdose and potentially fatal respiratory depression
Heroin	It is an illegal, very addictive opioid made from morphine	IV injection, IN, and inhalation (smoking)	hCE1 and hCE2 once formed morphine by UGT2B7 and other UGTs	P-gp	Increased risk of heroin overdose and the potential for suboptimal ARV efficacy

^*a*^ This table summarizes opioids commonly encountered in people living with HIV, including opioids used in clinical settings, medications for opioid use disorder (MOUD), and illicit opioids, with emphasis on pharmacological use, route of administration, metabolism, and possible clinically relevant interactions with antiretroviral drugs. PLWH, people living with HIV; OUD, opioid use disorder; IV, intravenous; IM, intramuscular; SC, subcutaneous; IN, intranasal; UGT, UDP-glucuronosyltransferase; CYP, cytochrome P450; BCRP, breast cancer resistance protein; OAT, organic anion transporter; OCT, organic cation transporter; P-gp, permeability glycoprotein.

## POTENTIAL DRUG-DRUG INTERACTIONS AND SIDE EFFECTS OF LONG-ACTING ANTIRETROVIRAL AND OPIOIDS

**Fig 1:**
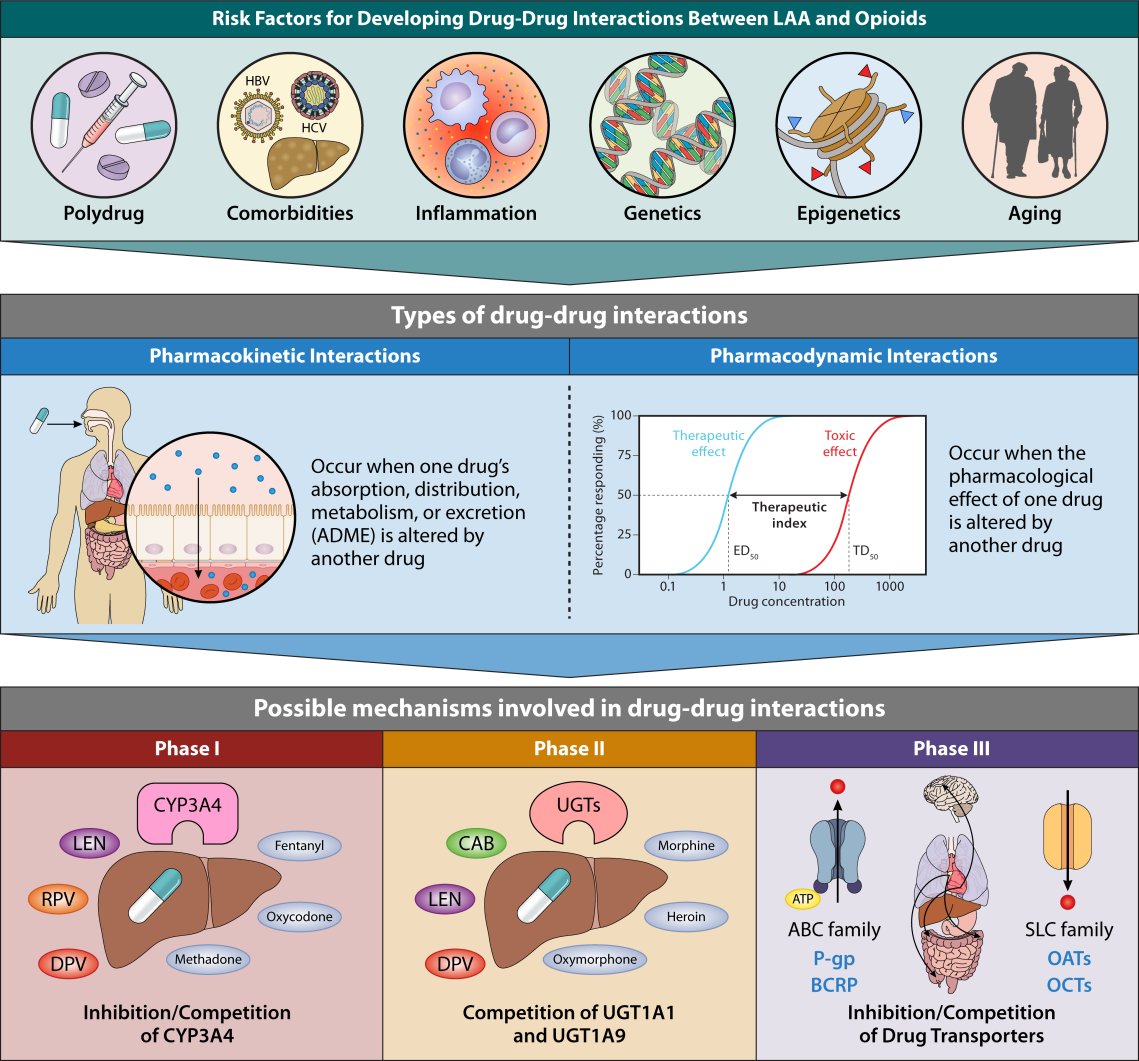
Risk factors and mechanisms for drug-drug interactions between long-acting antiretrovirals and opioids. The development of drug-drug interactions (DDIs) between long-acting antiretrovirals (LAAs) and opioids is influenced by several factors, including polydrug use, comorbidities, inflammation, genetic variations, and aging. DDIs can result from pharmacokinetic interactions, in which one drug affects the absorption, distribution, metabolism, or excretion (ADME) of another, as well as from pharmacodynamic interactions, in which the effects of one drug are altered by another. A key mechanism for these interactions involves the phase I metabolism mediated by the Cytochrome P450 enzyme CYP3A4, which is crucial for metabolizing both LAA (e.g., LEN, RPV, DPV) and opioids (e.g., fentanyl, oxycodone, methadone). Additionally, phase II metabolism can be influenced by uridine glucuronosyltransferases (UGT), particularly UGT1A1 and UGT1A9, which metabolize various LAAs (e.g., CAB, LEN, DPV) and opioids (e.g., morphine, heroin, oxymorphone). Phase III metabolism may also be impacted by competition among drug transporters, including P-glycoprotein (P-GP) and organic anion transporters (OATs), which are essential for the biodistribution of these medications. Understanding these interactions is vital for optimizing patient management and therapeutic outcomes.

**Fig 2:**
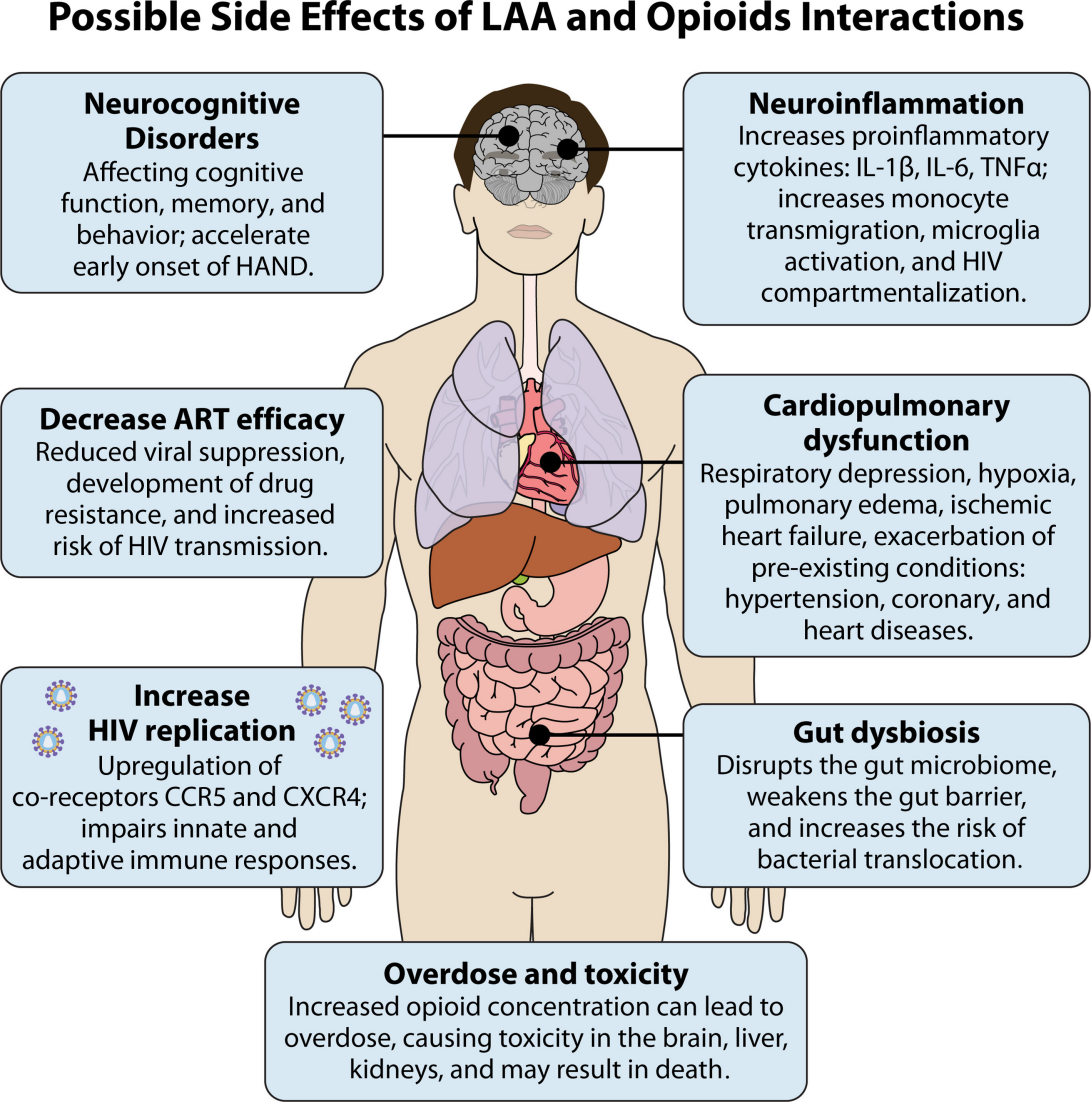
Potential side effects of long-acting antiretrovirals and opioid interactions. The interaction between long-acting antiretrovirals (LAAs) and opioids presents a range of possible adverse effects that necessitate careful consideration. The following are key potential side effects associated with this interaction: (1) neurocognitive disorders, (2) neuroinflammation, (3) reduced efficacy of antiretroviral therapy (ART), (4) cardiopulmonary dysfunction, (5) increased HIV replication, (6) gut dysbiosis, and (7) risk of overdose and toxicity. Given these potential risks, it is vital for healthcare providers to closely monitor and manage patients who are concurrently using LAA and opioids. A cautious approach to addressing these interactions is essential to ensure patient safety and optimize treatment outcomes. Abbreviations: LAA, long-acting antiretroviral; DDIs, drug-drug interactions; PK, pharmacokinetics; PD, pharmacodynamics; HIV, human immunodeficiency virus; AIDS, acquired immunodeficiency syndrome; PBPK, physiologically based pharmacokinetics; ADME, absorption, distribution, metabolism, excretion.

Drug metabolism is a complex physiological process that transforms drugs into more water-soluble compounds, facilitating their excretion and affecting their efficacy, safety, and duration of action. This process primarily occurs in the liver and mainly involves CYP450 and UGT enzymes (54). In the synergist HIV-OUD, DDIs could significantly influence the effects of LAA, opioids, and their metabolites (Fig. 1). A quicker elimination of any of these drugs may prevent them from reaching their therapeutic effect. Conversely, if they stay in the body for too long, it can cause cytotoxicity. Opioids have a narrow therapeutic index, and their metabolism produces both inactive and active metabolites, with some active forms possibly being more potent than the original drug (23, 27). Moreover, LAA introduces uncertainties due to prolonged drug levels after a single injection and the absence of a method to remove the medication. Therefore, while metabolism primarily serves as a critical detoxification process, being influenced by DDIs can disrupt the production of intermediate products, leading to important clinical outcomes, as well as present risks of toxicity and overdose (49, 55). Some of the possible side effects are explained in Fig. 2. Understanding the dynamic interaction between LAA and opioids is essential for optimizing these therapies and ensuring patient safety. Since LAA has recently been approved, there is currently limited or no data available on specific DDIs between these agents and opioids, such as morphine and fentanyl. Nonetheless, some clinical guidelines and the Liverpool drug interaction website (https://www.hiv-druginteractions.org/) mention the potential interaction between these medications. Furthermore, studies examining the interaction between previously approved antiretrovirals and opioids or other drugs that are substrates or inhibitors of CYP3A4, UGTs, and drug transporters may provide a valuable reference to underscore this concern and support the need for appropriate clinical precautions.

### Interactions affecting phase I of drug metabolism

Phase I metabolism transforms drugs by introducing or exposing polar functional groups through oxidation, reduction, or hydrolysis reactions, mainly catalyzed by the P450 enzyme system (54). CYP3A4 is the primary enzyme responsible for metabolizing fentanyl, oxycodone, tramadol, and methadone, and its substrates or inhibitors, including LAA, such as RPV, LEN, and DPV (Fig. 1). Research indicates that co-administration of opioids with other antiretrovirals that inhibit CYP3A4, such as protease inhibitors (PIs), could result in excessive opioid exposure (56). For instance, atazanavir or atazanavir/ritonavir has been linked to significant increases in levels of buprenorphine and its metabolite, as well as to adverse events related to sedation and cognitive impairment (57). It is essential to note that, currently, there are two available LAI buprenorphine products, which differ in dosing frequency, available dosages, and administration sites. Both are administered subcutaneously by a healthcare professional (10). Given that there is interest in combining these medications with LAA to provide an integral injectable treatment for people with comorbid HIV/OUD (58), clinicians should consider that CYP3A4 substrate/inhibitors─such as certain LAA (LEN, DPV)─can increase buprenorphine levels, thereby raising the risk of sedation and respiratory depression. Even some studies reported that DDI impact from CYP3A4 inhibitors is expected to be less significant with LAI compared to sublingual administration (59).

Moreover, Nieminen et al. reported that ritonavir and lopinavir/ritonavir significantly increased the plasma concentrations of oral oxycodone, thereby enhancing its effect in healthy volunteers (60). The authors suggest that when oxycodone is administered to patients receiving ritonavir and lopinavir/ritonavir, reductions in the oxycodone dose may be necessary to avoid opioid-related adverse effects. Also, a study by Olkkola et al. found that ritonavir reduced the clearance of fentanyl by 67% (61). Additionally, a PK study by Cambic et al. utilized human, mouse, rabbit, and feline models to simulate the effects of fentanyl in the absence and presence of ritonavir. The study showed that simulated plasma fentanyl concentrations in the ritonavir group were higher than those in the control group for all sample labor analgesic regimens. Also, fentanyl clearance was compromised, implying that fentanyl plasma concentration gradually decreased following an intravenous bolus and that accumulation of fentanyl may occur during infusion when it is administered alongside ritonavir (62).

LEN is a substrate and a moderate inhibitor of CYP3A4. Therefore, when administered alongside opioids metabolized by this enzyme, it could increase opioid concentrations, thereby prolonging and intensifying the analgesic and adverse effects of opioids in the brain, liver, kidneys, and immune system, while also increasing the risk of respiratory depression, overdose, and death. Additionally, the concomitant use of opioids can alter LEN metabolism and potentially affect its efficacy and safety. Furthermore, in 2024, the Liverpool HIV drug interaction website published a list of possible interactions of LEN with 1,073 diverse medications. It highlighted specific concerns about the use of opioids such as fentanyl and alfentanil due to the risk of increased opioid exposure, which may persist even after discontinuing LEN because of its residual CYP3A4 inhibitory effects. Therefore, prescribers should be aware that residual concentrations of LEN could remain in the systemic circulation for up to 12 months. Another important consideration is the initiation phase of LEN, which involves a course of oral tablets that may increase the risk of DDIs due to the moderate inhibition of CYP3A4 by LEN.

DPV is a substrate and a moderate inhibitor of CYP3A4. Consequently, DPV could lead to interactions with opioids, whose primary metabolites are metabolized by this enzyme, such as fentanyl, oxycodone, and methadone, among others. There is no data available regarding the DDIs of DPV, especially the vaginal ring and opioids. However, research showed that the concomitant use of a DVR and other CYP3A4 inhibitors, such as the antifungal miconazole (vaginal capsule), was found to increase systemic exposure to DPV by 20% compared to using a DVR alone (49). That is expected, since these two medications are administered intravaginally. Nonetheless, several studies have reported that DPV does reach the bloodstream and is present in plasma, breast milk, and various organs. A survey of the safety and PK trial of a DVR reported that the median DPV concentrations were 676 pg/mL in breast milk and 327 pg/mL in plasma (48). In addition, research in animal models has shown detectable concentrations of DPV in liver tissue following vaginal administration. While the exact liver concentrations differ across studies and dosages, it is generally observed that DPV concentrations can be detected in the liver from day 1 of DPV exposure (63). Human studies, on the other hand, have focused on plasma and cervical tissue concentrations, with less emphasis on specific organs such as the liver. However, some studies have investigated the impact of DPV on liver enzymes and metabolism. The distribution of DPV across different tissues is influenced by factors such as the drug’s lipophilicity and the presence of DDIs that affect drug-metabolizing enzymes. Although most clinical trials indicate that DPV satisfactorily meets efficacy and safety standards, with no significant safety concerns, some studies have been shorter. For example, a 13-week study, the longest being a 24-month phase III trial, focused mainly on efficacy rather than on the development of interactions (14, 64). Therefore, further research is recommended to study the long-term effects of DPV on the development of drug interactions, especially with opioids, and cytotoxic effects on the liver, brain, and other organs.

RPV is primarily metabolized by CYP3A4, but it does not inhibit or induce CYP3A4 at clinical doses. This means that RPV is less likely to interact with other drugs that are CYP3A4 substrates, as in the case of opioids and related medications, previously described. However, as RPV and these drugs are both substrates of CYP3A4, pharmacological (PK, PD) interactions could occur. Crauwels et al. reported that the coadministration of once-daily oral RPV (25 mg) and methadone (60–150 mg) decreased the AUC of methadone by 16% (65). The authors noted that no *a priori* dose adjustment of methadone was needed. However, clinical monitoring is recommended, as methadone maintenance therapy could require adjustments in some patients. A recent case series examines the 2-year implementation of CAB/RPV in PLWH with OUD. The study observed 100% adherence and 100% viral suppression among all 15 patients. In terms of adverse outcomes, one patient died during the study period due to an opioid overdose after the patient resumed engaging in substance use (66). The authors state that this death was not related to the administration of CAB/RPV. However, this underscores the need for continuous monitoring of these sensitive populations to minimize risks and ensure safe, successful therapy.

Opioids, like all CNS depressants, suppress the respiratory system. Data indicate that the complex interplay between the metabolism of LAA medications, which are substrates and/or inhibitors of CYP3A4, and opioids that use the same enzymatic system could result in interactions. These DDIs could significantly increase the concentrations of fentanyl, oxycodone, codeine, methadone, and buprenorphine, leading to overdose, inducing hypoventilation, damaging various organs, and even causing death, as is explained in Fig. 2. Given the limited studies on the interaction between LAA and opioids, the clinical relevance of these potential interactions cannot yet be established. Future clinical trial results will inform forthcoming guidelines. In the interim, clinicians should be advised to inform and educate patients about the potential risks associated with LAA and poly-substance use, particularly when illicit drugs, such as fentanyl and its derivatives, are involved.

### Interactions affecting phase II of drug metabolism

Phase II metabolism, or conjugation, is a crucial part of drug metabolism, where the body adds polar groups to the drug or its metabolite. These reactions generally involve enzymes called transferases that attach a polar group (like glucuronic acid, sulfate, or glutathione) to the molecule. The UGT is a secondary metabolizing system responsible for forming glucuronides. UGT enzymes are the primary enzymes responsible for CAB metabolism, mainly UGT-1A1, with minor contributions from UGT-1A9. LEN and DPV have mixed metabolic pathways, including CYP3A4 and UGT1A1. The UGTs, especially UGT1A1, 1A3, 1A6, 8, 1A9, and 2B7, have a significant role in the metabolism of opioids, such as morphine, heroin, hydromorphone, and oxymorphone. Competitive and non-competitive interactions between substrates of UGTs can occur when two or more substances are metabolized by the same UGT enzyme (Fig. 1). These interactions could increase opioid concentration in the bloodstream, brain, liver, and kidneys. If LAA inhibits UGTs, it could lead to higher levels of some opioids, potentially increasing their effect or causing adverse effects, as explained in Fig. 2. Conversely, if LAA reduces UGT activity, it could lead to lower levels of some opioids, potentially reducing their effectiveness. Also, LAA metabolism could be affected.

Real-world studies confirm that CAB demonstrates high effectiveness, with viral suppression rates exceeding 99% in PrEP, and it offers notable convenience by decreasing the daily pill burden. However, these studies also show variability in drug concentration levels even when using the exact dosage and route of administration (40). One possible explanation for these variations is DDIs that accumulate through hepatic metabolism (28). The competition of CAB for the UGT enzymes has been reported with other substrates. Various studies that evaluated the safety, tolerability, and side effects of CAB found that some patients presented elevations in ALT, AST, and bilirubin. This situation was described as secondary to competition between cabotegravir and unconjugated bilirubin for elimination via UGT1A1 (67, 68). Previous ARVs, such as the protease inhibitor atazanavir, have been associated with inhibiting the UGT1A1 enzyme, which prevents the glucuronidation and elimination of bilirubin. This can cause indirect hyperbilirubinemia with jaundice, potentially leading to early discontinuation of atazanavir (69–71). Adverse events related to bilirubin elevations, such as hyperbilirubinemia, jaundice, and scleral icterus, should be monitored, as they can be signs of possible DDIs or other forms of drug-induced liver injury.

Recent evidence also shows that inflammation, a key feature in PLWH, downregulates the expression and activity of specific UGT enzymes, particularly major isoforms, such as UGT1A1, UGT1A9, and UGT2B (72–74). The downregulation is mainly caused by pro-inflammatory cytokines and can have important clinical effects on drug metabolism and the clearance of endogenous compounds. This may potentially increase the exposure and toxicity of LAA and opioids that are metabolized through glucuronidation, such as CAB, morphine, codeine, and hydrocodone. DPV is also a moderate inhibitor of UGT1A1 and a weak inhibitor of UGT1A9 and UGT2B7, which could lead to possible DDIs with these opioids (49).

Interactions involving class II metabolism enzymes may also arise when two different opioids are co-administered. Methadone has been shown to significantly increase morphine plasma concentrations and reduce morphine clearance *in vivo* (75). Additionally, methadone decreases the formation of morphine-3-glucuronide and morphine-6-glucuronide, with the reduction in morphine-6-glucuronide formation being less pronounced than that of morphine-3-glucuronide. This inhibition is attributed to the effects of both R- and S-methadone on the UGT2B7 enzyme (76).

Limited studies have examined interactions of opioids and LAA involving phase II metabolic enzymes. The recommendations in this review are based on theoretical predictions based on known competitive or inhibitory effects from other drugs, such as previously approved ARVs. Further research is necessary to determine the clinical relevance of these possible DDIs.

### Interactions affecting phase III of drug metabolism

Phase III of drug metabolism involves specialized transporters that move these conjugates and metabolites out of cells and into the bloodstream, bile, or urine (77). Drug transporters are integral membrane proteins expressed in various tissues that play a crucial role in maintaining the integrity of physiological barriers, including the blood-brain barrier (BBB) (78). There are two major superfamilies of transporters: ATP-binding cassette (ABC) transporters and solute carrier (SLC) transporters (79). ABC transporters include the permeability glycoprotein (P-gp), breast cancer resistance protein (BCRP), and multidrug resistance-associated proteins (MRPs) (80). Among the primary transporters of the SLC superfamily are the organic anion transporters (OATs) and the organic cation transporters (OCTs) (81, 82). It is important to note that opioid agonist treatment is also abbreviated (OATs). This review strictly uses OATs to refer to organic anion transporters to avoid confusion.

ABC and SLC transporters play a critical role in the metabolism of many drugs, including opioids, their metabolites, and endogenous compounds such as LAA, by promoting drug absorption, distribution, and elimination (Fig. 1). Research indicates that transporters in the gut and liver influence opioid bioavailability by regulating how much of an opioid enters the body and how much escapes first-pass metabolism (83). Additionally, transporters in the liver and kidneys facilitate the excretion of opioids and their metabolites into bile or urine. Transporters at the BBB influence the movement of opioids into and out of the brain, affecting their analgesic and other effects. P-gp, for example, can reduce the brain concentration of opioids, potentially impacting their efficacy (84). Transporters, such as OCT, are involved in transporting opioids into liver cells for metabolism, as demonstrated by Tzvetkov et al., who reported that OCT1 plays a crucial role in the hepatocellular uptake of morphine (85).

Furthermore, multiple studies have reported that LAA can function as substrates and/or inhibitors of drug transporters. CAB is a substrate of P-gp and BCRP, an inhibitor of OAT1 and OAT3 (32). LEN is a P-gp substrate, inhibiting P-gp and BCRP (41). Additionally, RPV can inhibit P-gp, BCRP, and OAT1 (86). Conversely, DPV is neither a substrate nor an inhibitor of MRP, P-gp, or BCRP transporters (87). Since most opioids are substrates for drug transporters, LAA could be both a substrate and/or an inhibitor of the same transporters. Aside from drug-metabolizing enzymes, drug transporters could contribute to DDIs when both opioids and LAA are used concomitantly. These interactions are therefore critical determinants of the PK of these drugs that could alter the pharmacological effects and induce overdose.

CAB is a substrate of P-gp and BCRP; however, data indicate that the influence of these transporters on CAB disposition is expected to be minimal, given the compound’s high passive membrane permeability. Nonetheless, morphine, fentanyl, and codeine are substrates of these same transporters; therefore, pharmacological evaluations should consider this interaction when CAB and opioids are used together. Specifically, the long pharmacokinetic tail of CAB highlights its clinical significance, as it indicates the post-injection duration during which the drug remains detectable in the body, potentially lasting several months (88). This characteristic has essential ramifications regarding the risks of HIV infection, DDIs, and resistance once dosing has stopped. Clinical trials have estimated that, in some cases, the concentration of CAB may remain above the limit of quantitation for as long as 4.3 years in females and 2.9 years in males, with longer durations occurring in individuals with higher BMI (89). Additionally, CAB and RPV can also affect opioid metabolism by inhibiting OATs. A report by Reese et al. demonstrated that CAB inhibited OAT1 and OAT3 *in vitro*. Consequently, the clinical impact is considered a concern for substrates of OAT1 and OAT3, such as buprenorphine, hydrocodone, M6G, and C6G (32). This can lead to increased exposure to opioids or alterations in their clearance, requiring dose adjustments or careful monitoring.

LEN is a substrate of P-gp and, more importantly, an inhibitor of P-gp and BCRP (90). Several opioids are known to be substrates of P-gp, which is an efflux transporter that can limit their penetration into the brain. These include morphine, fentanyl, the MOUD, methadone, and potentially others (91). Inhibiting P-gp can lead to increased opioid concentrations in the brain and plasma, potentially enhancing analgesic and other effects while also increasing the risk of toxicity (30). Studies have shown that when P-gp is inhibited, fentanyl is less effectively transported out of cells, leading to increased risks of neurotoxicity, respiratory depression, and sedation (30, 92). Moreover, inhibiting P-gp can elevate morphine concentrations in the brain, potentially raising the risk of overdose. Experimental studies have shown that the co-administration of morphine with a P-gp inhibitor could alter the systemic PK and antinociceptive PD of morphine by enhancing morphine antinociception and elevating systemic M3G concentrations (25, 93). It is also essential to be aware of the potential DDIs when co-administering morphine with other P-gp inhibitors, such as LEN and RPV.

Furthermore, LEN and RPV are substrates and/or inhibitors of BCRP, which plays a critical role in opioid metabolism, particularly at the BBB and in placental tissue. BCRP acts as an efflux transporter, suggesting that it moves drugs and other substances out of cells and tissues. In the context of opioids, BCRP can help limit the entry of opioids into the brain and placenta, potentially influencing their analgesic effects and reducing fetal exposure to opioids (94). In a report by Neradugomma et al., the authors stated that buprenorphine, norbuprenorphine, and methadone significantly increased the expression of BCRP mRNA up to 10-fold in human cells (95). Given the critical role of BCRP in limiting fetal exposure to drugs and xenobiotics, DDIs mediated by competition or inhibition of these transporters could affect fetal drug exposure in the human placenta. Overall, these data suggest that drug interactions affecting the third phase of metabolism may impact the success of HIV treatment, control of opioid dependence, and increase the risks of toxicity and overdose. Therefore, further research is necessary to determine their clinical effects and ensure careful monitoring and management. Additionally, people with co-occurring HIV and OUD face a complex interplay of health and social challenges, requiring an integrated and person-centered approach to care. This includes harm- and risk-reduction interventions, developing actionable strategies to reduce stigma, and providing education on safer injection practices (96, 97).

## FACTORS INCREASING THE RISK OF DRUG-DRUG INTERACTIONS AND CLINICAL CONSIDERATIONS

### Aging and polypharmacy

PLWH face a higher risk of accelerated aging and the onset of comorbidities. Consequently, alongside ART, they require additional medications and are at a higher risk for DDIs. Age-associated physiological changes that alter PK and PD include increased adiposity, decreased albumin levels, and changes in the expression and function of metabolic enzymes (98). Studies have shown that the expression and function of CYP 450, UGT, and drug transporters decrease in older individuals (99–101). Ximenes et al. reported that intestinal P-gp activity decreased by about 40% in postmenopausal individuals. This age-related reduction could lead to increased plasma exposure of P-gp substrates, such as opioids and LAA (102). There is limited data about the PK of LAA because they have been poorly studied in older PLWH and are often excluded from clinical trials. A recent report indicates that LA CAB/RPV concentrations are expected to be higher in older people than in younger individuals. This raises concern because it could lead to increased drug-related toxicity and drug interactions in this group (103). In addition, PBPK models showed that aging led to a modest increase in the exposure of oral CAB (AUC increased by 20%) and RPV (AUC increased by 25%). Similarly, aging was predicted to have a modest effect on intramuscular CAB/RPV PK after monthly or bimonthly administration (104).

### Comorbidities include inflammation, hepatitis B (HBV), hepatitis C (HCV), hepatic damage, and renal disease

Chronic inflammation caused by persistent immune activation, microbial dysbiosis, and co-infections is notable comorbidities in PLWH (105–107). Inflammation can lead to downregulation of CYP450, UGTs, and P-gp (108). In a study by Zeng et al., it was reported that inflammatory milieu downregulated the expression of UGT1A1 and UGT1A9 in the mouse liver, which is essential for the glucuronidation of CAB. Peripheral inflammatory pain causes a redistribution of P-gp and increases P-gp activity, decreasing the accumulation of morphine in the brain (109).

Liver impairment caused by Hepatitis B, Hepatitis C, and certain ART-related hepatotoxicity can alter hepatocyte function, reducing their ability to produce and utilize CYP and UGT enzymes. A recent study by Hanked et al. has revealed a decline in the expression and activity of CYP3A4, along with changes in influx and efflux transporters such as OAT, in patients with liver disease (110, 111). Clinical trials showed that some patients receiving CAB may experience elevated alanine aminotransferase (ALT) and aspartate aminotransferase (AST) (112). Also, research indicates that in patients with moderate to severe hepatic impairment, the PK exposure of LEN, such as AUC and Cmax, was higher compared to controls (9).

Renal diseases pose a significant concern for PLWH since HIV can affect kidney function by damaging the glomeruli and tubules, and individuals with pre-existing conditions may be at higher risk of complications during ART (113, 114). Moreover, certain ART can contribute to the renal impairment directly, through nephrotoxicity, and indirectly, due to potential DDIs (115, 116). People with chronic inflammation or hepatic and renal impairment could be affected by DDIs between LAA and opioids, as this phenomenon can impair drug biodistribution, metabolism, and elimination, potentially leading to increased exposure, toxicity, and overdose.

### Genetic and epigenetic modifications lead to drug-drug interactions 

Genetic and epigenetic factors play crucial roles in how individuals respond to drug treatments, influencing drug metabolism and overall therapeutic outcomes (117). Genetic polymorphisms in drug-metabolizing enzymes can significantly influence the rate of drug metabolism and elimination, potentially affecting both efficacy and safety profiles. Polymorphic CYP450 and UGT enzymes can play key roles in differential drug efficacy and toxicity among various individuals. Mutations in CYP3A4, UGT1A1, and UGT2B involve numerous genetic variations, including single-nucleotide polymorphisms (SNPs), which can lead to abolished, reduced, altered, or increased enzymatic activity (118). Currently, numerous SNPs have been identified in CYP3A4. These polymorphisms categorize the general population into poor metabolizers, normal metabolizers, and rapid metabolizers. It is well known that genetic variation, not only in CYP3A4 but also in other CYP450 enzymes, can lead to significant variations in serum levels of opioids, such as codeine, fentanyl, methadone, oxycodone, and oxymorphone. Consequently, the FDA has approved a genotype test that physicians can use to select medications metabolized by CYP450 enzymes. But in clinical practice, their use is limited by high cost. However, in the near future, their use is expected to increase, and they could be included in health insurance plans, as studies demonstrate their cost-effectiveness (119). In addition, polymorphisms within the ABCB1 gene, particularly SNPs, could alter P-gp activity and potentially influence drug efficacy and its side effects. On the other hand, epigenetic changes can affect gene expression without altering the DNA sequence, further modulating drug response (120). DNA methylation, histone modifications, and microRNAs are examples of epigenetic mechanisms that can alter gene expression (121). An animal study by Kalscheuer et al. demonstrated that inducing DNA methylation reduced the expression of several microRNAs, including miRNA-126. This change led to increased expression of CYP2A3 in rat lungs, similar to that of CYP3A4 in humans (122). Research conducted on human subjects has shown a negative correlation between low UGT1A1 expression levels and the extent of gene promoter methylation (123). Consequently, when LAA and opioids are prescribed together, genetic and/or epigenetic modifications should be considered as factors that could influence drug efficacy, toxicity, and even susceptibility to diseases.

## POTENTIAL AVENUES FOR INVESTIGATING THE DRUG-DRUG INTERACTIONS BETWEEN LONG-ACTING ANTIRETROVIRAL AND OPIOIDS

**Fig 3:**
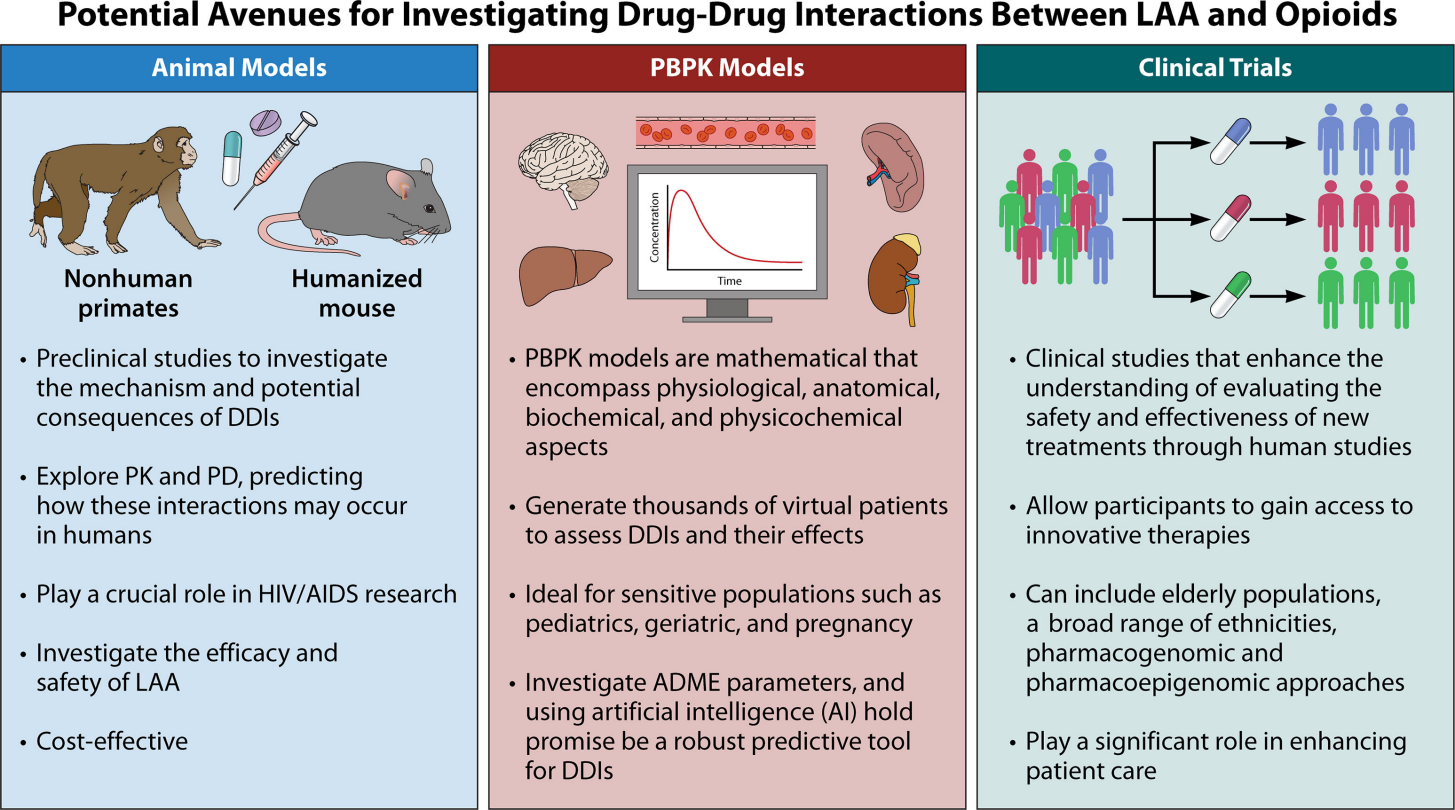
Potential avenues for investigating drug-drug interactions between long-acting antiretroviral and opioids. This illustration presents complementary experimental, translational, and clinical approaches for assessing pharmacokinetic and pharmacodynamic interactions between long-acting antiretroviral agents and opioids. These approaches include animal models, physiologically based pharmacokinetic (PBPK) models, and clinical trials, each summarized with their main characteristics and advantages.

Various avenues, including animal models, physiologically based pharmacokinetic (PBPK) studies, and clinical trials, could be used to evaluate the safety and efficacy of new treatments, such as LAA, alongside opioids in PLWH. A summary of the main characteristics of these approaches is shown in Fig. 3. These models provide critical insights into the PK and PD of these interactions, assisting in forecasting how they might manifest in humans or be extrapolated to other populations. Animal models, particularly non-human primates and humanized mice, play a crucial role in HIV/AIDS research due to their close resemblance to human disease progression (124–128). PBPK models are mathematical models that encompass physiological, anatomical, biochemical, and physicochemical parameters to describe ADME of xenobiotics and their metabolites (129). PBPK model has become an essential tool in drug discovery and development and is now widely accepted by regulatory authorities for assessing CYP450, UGT, and transporter-mediated DDIs (130, 131). Clinical trials are crucial to the development of new medical interventions and significantly enhance patient care (132). Clinical trials also contribute to a deeper understanding of diseases and the development of more effective treatments for future patients (133, 134). Therefore, the inclusion of elderly populations, a broad range of ethnicities, pharmacogenomic and pharmacoepigenomic approaches, as well as vulnerable groups—such as those with OUD or renal/hepatic impairments—is necessary to investigate possible drug interactions and their consequences on treatment efficacy and toxicity between LAA and opioids.

## CONCLUSION AND FUTURE DIRECTIONS

LAAs have emerged as a preferred and successful alternative for HIV treatment and prevention, particularly among individuals facing adherence challenges, such as those with OUD. DDIs are highly prevalent in PLWH, especially given the increasing development of comorbidities and polypharmacy. Cytochrome P450, especially CYP3A4, and UGT enzymes metabolize many drugs, including opioids and LAA. Drug transporters, such as P-gp, involved in phase III metabolism, also affect drug distribution. Interactions affecting drug metabolism of opioid and LAA may affect the success of HIV treatment, the control of opioid dependence, and the risk of toxicity and overdose. Although most LAAs do not seem to significantly interfere with major metabolic enzymes or transport pathways, co-administration with drugs that have a narrow therapeutic index, such as opioids, can still be affected by certain physiological and clinical conditions that influence their effectiveness and side effects. Factors such as aging, liver/kidney dysfunction, and inflammation should be considered in clinical practice. Limited studies have explored interactions between opioids and LAA. Evidence from pharmacodynamic and pharmacokinetic studies, including PBPK models and clinical trials, remains scarce. Some recommendations in the Liverpool HIV Interaction database guidance and in this review are based on theoretical predictions derived from known induction or inhibition effects of other drugs, such as previously approved ARVs. It is imperative to determine the significance of these possible DDIs across different patient groups and provide clear guidance to healthcare providers and patients. People with co-morbid HIV and OUD face complex health and social challenges. Consequently, it is essential to offer personalized, holistic support that aligns with their specific circumstances. This includes harm reduction strategies, efforts to reduce stigma, and education about safer injection practices.
